# Lactate Aggravates MASLD via PPARγ/CD36-Mediated Hepatocellular Fatty Acid Uptake

**DOI:** 10.3390/cells15141240

**Published:** 2026-07-09

**Authors:** Wenke Sun, Weiwei Li, Guangyi Ouyang, Jishuang San, Yue Zhu, Yunheng Liu, Jiancheng Yang, Gaofeng Wu

**Affiliations:** College of Animal Science and Medicine, Shenyang Agricultural University, No. 120, Dongling Road, Shenyang 110866, China; swkjsmh666@stu.syau.edu.cn (W.S.); liweiwei93@syau.edu.cn (W.L.); ouyangguangyi2022@163.com (G.O.); sanjishuang@syau.edu.cn (J.S.); 15040935083@163.com (Y.Z.); 2024200210@stu.syau.edu.cn (Y.L.)

**Keywords:** MASLD, lactate, PPARγ/CD36 signal pathway, AAV8-TBG-shRNA, network pharmacology

## Abstract

**Highlights:**

**What are the main findings?**

- Hepatic LDH is significantly upregulated in MASLD, and lactate exacerbates hepatic lipid accumulation.
- Lactate-induced hepatosteatosis is mediated by the PPARγ/CD36 axis; silencing LDHA, PPARγ or CD36 attenuates this phenotype.

**What are the implications of the main findings?**

- A novel mechanistic link between aberrant lactate buildup and MASLD progression is identified.
- The PPARγ/CD36 axis represents a promising therapeutic target for MASLD intervention.

**Abstract:**

Background: Metabolic dysfunction-associated steatotic liver disease (MASLD) is now the most prevalent chronic liver disease worldwide, imposing a severe public health burden. Its core pathological hallmark is excessive hepatic lipid accumulation driven by systemic metabolic dysregulation. Concomitant hepatocellular injury impairs hepatic lactate clearance, leading to aberrant lactate buildup in the liver microenvironment. However, the causal role of lactate in exacerbating liver lipid metabolism dysfunction and driving the progression of MASLD remains unclear. Methods: First, we performed a comprehensive bioinformatic analysis of publicly available transcriptomic datasets. Mining of the Gene Expression Omnibus (GEO) database showed that lactate dehydrogenase (LDH) expression was significantly upregulated in liver tissues from both metabolic dysfunction-associated fatty liver disease (MASLD) patients and MASLD mouse models. Next, network pharmacology approaches were employed to predict putative molecular targets that could mediate lactate’s biological effects. Gene Ontology (GO) and Kyoto Encyclopedia of Genes and Genomes (KEGG) enrichment analyses indicated that these candidate targets were predominantly enriched in pathways governing fatty acid metabolism and long-chain fatty acid transport. Molecular docking and molecular dynamics simulations further suggested possible interactions and supported the prioritization of cluster of differentiation 36 (CD36) as candidate lipid metabolism regulators potentially involved in lactate-mediated effects. Finally, liver-specific Ldha knockdown mice (AAV8-TBG-shRNA) and free fatty acid-induced steatotic AML12 hepatocytes were used to investigate the functional relevance of these findings in vivo and in vitro. Results: Network pharmacology analyses preliminarily identified the PPAR signaling pathway as a candidate pathway potentially linking lactate to MASLD. Experimental results showed that exogenous lactate administration was associated with significantly increased lipid accumulation in steatotic AML12 hepatocytes and the livers of MASLD mice, manifested as elevated triglyceride levels and enhanced lipid droplet formation, accompanied by upregulated expression of PPARγ and CD36. Conversely, inhibiting endogenous lactate production or silencing PPARγ or CD36 attenuated this lipid-accumulation phenotype and significantly reduced intracellular triglyceride levels. Conclusions: In conclusion, these findings indicate that lactate exposure is associated with hepatic lipid accumulation and upregulation of the PPARγ/CD36 axis. Pharmacological inhibition or silencing of PPARγ or CD36 attenuates this phenotype, suggesting that this pathway may contribute to lactate-associated hepatic steatosis and potentially accelerate MASLD progression.

## 1. Introduction

Metabolic Dysfunction-Associated Steatotic Liver Disease (MASLD), formerly known as Nonalcoholic Fatty Liver Disease (NAFLD), is a chronic liver disorder officially renamed at the 2023 International Consensus on Fatty Liver Disease [1,2,3,4,5]. A recent meta-analysis reported a global adult MASLD prevalence of 29.8%, affecting over 1 billion individuals worldwide, with prevalence exceeding 30% in China and continuing to rise. MASLD and its progressive complications account for approximately 2 million liver-related deaths annually, imposing a rapidly growing global healthcare burden. The pathological progression of MASLD encompasses multiple sequential stages, with a highly complex pathogenesis involving multiorgan dysregulation and pathway perturbations. It is well established that dysregulation of hepatic lipid metabolism constitutes the core initiating event in MASLD pathogenesis and the key driver of the progression from simple steatosis to steatohepatitis, liver fibrosis, and ultimately cirrhosis. Notably, MASLD has emerged as the leading driver of the rising global incidence of hepatocellular carcinoma (HCC), even in the absence of cirrhosis.

Despite this substantial disease burden, effective pharmacotherapies for MASLD have long been scarce. In 2024, resmetirom received accelerated FDA approval as the first licensed therapy for patients with noncirrhotic MASH and moderate to advanced fibrosis, marking a milestone in MASLD clinical management. Nevertheless, treatment options remain very limited, and in-depth dissection of the molecular mechanisms driving MASLD progression is critical for the development of additional, more effective therapeutic strategies.

Lactate (LA), the end product of glycolysis, was long considered merely a metabolic waste prior to the elucidation of the Warburg effect. However, advances including the lactate shuttle theory and the discovery of protein lactylation have reshaped this paradigm: lactate is now established as both a core energy substrate and a multifunctional signaling and epigenetic regulator [6]. Multiple clinical studies have documented significantly elevated lactate levels in both liver tissue and peripheral blood of patients with MASLD compared with healthy controls [7,8], with lactate accumulation positively correlated with the severity of steatosis, hepatic inflammation, and fibrosis stage.

Mechanistically, pathological features of MASLD such as systemic insulin resistance and enhanced hepatic glycolytic flux jointly increase hepatic lactate production while impairing lactate clearance, leading to pathological intrahepatic lactate accumulation. In turn, accumulated lactate exacerbates hepatic lipid dysregulation, inflammasome activation, and hepatic stellate cell activation, forming a vicious cycle of metabolic dysfunction, lactate buildup, and progressive liver injury. For instance, lactate has been shown to activate the mitogen-activated protein kinase (MAPK) pathway, induce pro-inflammatory cytokines (IL-6, TNF-α) and oxidative stress, and thus aggravate hepatocellular injury and fibrosis, which is associated with adverse clinical outcomes in MASLD. In MASH mouse models, lactate drives hepatic metabolic remodeling and upregulates key glycolytic enzymes including phosphoglucomutase (PGM) and phosphoglucose isomerase (PGI), creating a positive feedback loop that promotes MASH progression and predisposes to cirrhosis [9,10]. Furthermore, enhanced lactate production via the Warburg effect in early hepatic steatosis is linked to subsequent inflammation, fibrosis, and elevated HCC risk [11,12,13].

Building on the aforementioned research background, this study aimed to elucidate the role and underlying mechanism of lactate in hepatic lipid metabolism during early-stage metabolic dysfunction-associated steatotic liver disease (MASLD) by integrating bioinformatic prediction with in vitro and in vivo functional validation. We identified the PPARγ/CD36 axis as a key mediator of lactate-driven hepatic lipid accumulation and confirmed that targeting endogenous lactate production or the PPARγ/CD36 pathway attenuates lactate-induced hepatic steatosis. Collectively, this study provides a novel perspective on MASLD pathogenesis and lays a foundation for future mechanistic and translational research targeting lactate metabolism.

## 2. Materials and Methods

### 2.1. Data Acquisition and Preliminary Processing

Publicly available liver tissue transcriptome datasets were retrieved from the Gene Expression Omnibus (GEO) database (https://www.ncbi.nlm.nih.gov/geo/, accessed on 8 May 2025) to validate the expression levels of lactate dehydrogenase isoforms (*LDHA* and *LDHB*) in MASLD.

Dataset inclusion criteria were defined as follows: (1) human liver tissue samples; (2) high-throughput RNA sequencing data; (3) histologically confirmed MASLD diagnosis with healthy controls; (4) complementary cohort characteristics in terms of age and ethnicity.

Three independent datasets were finally included, covering both human clinical samples and mouse disease models:

- The GSE185051 dataset was set as the primary validation cohort, which contains liver biopsy samples from 52 pediatric MASLD patients and 5 healthy controls with multi-ethnic background (71% Hispanic), profiled by Illumina HiSeq 2000 platform. The large sample size ensures sufficient statistical power for differential expression analysis;
- The GSE260666 dataset was included as an independent cross-validation cohort. It is a Chinese adult cohort consisting of 6 patients with simple steatosis (NAFL), 4 patients with steatohepatitis (NASH) and 6 healthy controls. This cohort precisely matches the disease stage focused in our study and the local population background, which is used to verify the consistency of findings across different age groups and ethnicities;
- GSE205021 (mouse, model validation cohort): 12 liver samples in total (6 normal diet controls, 6 high-fat diet-induced MASLD models), used for cross-species validation of the expression pattern.

Raw sequencing reads were first subjected to quality control using FastQC (v0.11.9) to assess base quality, GC content, and adapter contamination. Clean reads were then aligned to the human reference genome (GRCh38.p13) for human datasets or the mouse reference genome (GRCm39) for the mouse dataset using the STAR aligner (v2.7.10a) with default parameters. Gene-level read counts were quantified using featureCounts (v2.0.3) from the Subread package. Differential expression analysis was performed using the DESeq2 R package (v1.38.3). Raw read counts were normalized using the median of ratios method, and differential expression statistics were calculated using the Wald test. Genes with |log_2_ fold change (FC)| > 1 and false discovery rate (FDR)-adjusted *p*-value < 0.05 were considered significantly differentially expressed. The relative expression levels of LDHA and LDHB were visualized as box plots (showing median, interquartile range, and minimum/maximum values) using the ggplot2 R package (v3.4.0).

### 2.2. Prediction of Lactate Targets

Lactate-related targets were systematically identified by integrating data from multiple complementary databases to minimize bias and improve prediction reliability. The following four databases were queried using the SMILES structure of L-lactate (SMILES: CC@HC(=O)[O-]) as the input: Therapeutic Target Database (TTD) (https://db.idrblab.net/ttd/, accessed on 8 May 2025); Traditional Chinese Medicine Systems Pharmacology Database and Analysis Platform (TCMSP) (https://tcmsp-e.com/disease.php?qd=77, accessed on 8 May 2025); PharmMapper Server (http://lilab-ecust.cn/pharmmapper/, accessed on 8 May 2025); ChEMBL Database (https://www.ebi.ac.uk/chembl/, accessed on 8 May 2025).

For each database, only targets with a confidence score ≥ 0.7 (or equivalent threshold as defined by the respective database) were retained. All retrieved targets were converted to official gene symbols using the NCBI Gene database (https://www.ncbi.nlm.nih.gov/gene/, accessed on 8 May 2025) to ensure consistency across databases. Duplicate targets were removed by merging entries with identical gene symbols, retaining the highest confidence score for each unique target.

### 2.3. MASLD and Liver Injury-Related Target Acquisition

Disease-associated targets were retrieved using the keywords “non-alcoholic fatty liver disease”, “simple fatty liver disease”, “non-alcoholic steatohepatitis”, and “liver injury” in the following six databases: GeneCards (https://www.genecards.org/, accessed on 27 July 2025); Online Mendelian Inheritance in Man (OMIM) (https://www.omim.org/, accessed on 27 July 2025); DisGeNET (https://www.disgenet.org/, accessed on 27 July 2025); PharmGKB (https://www.pharmgkb.org/, accessed on 27 July 2025); DrugBank (https://go.drugbank.com/, accessed on 28 July 2025); NCBI Gene (https://www.ncbi.nlm.nih.gov/gene/, accessed on 28 July 2025).

Disease target selection criteria: For GeneCards, only targets with a relevance score ≥ 5.0 were included. For DisGeNET, only associations with a GDA score ≥ 0.3 were retained. All disease targets were standardized to official gene symbols.

### 2.4. Protein–Protein Interaction (PPI) Network Construction

The intersection of lactate-related targets and MASLD/liver injury-related targets was determined using the Venny 2.1 online tool (https://bioinfogp.cnb.csic.es/tools/venny/, accessed on 3 August 2025). These common targets were imported into Cytoscape 3.8.0 to construct a lactate-target-disease regulatory network. For PPI prediction, the common targets were submitted to the STRING Functional Protein Linkage Network database (https://string-db.org/, version 12.0, accessed on 3 August 2025) with the following parameters: Minimum required interaction score: medium confidence (≥0.4). The filtered PPI data were saved as tab-separated value (TSV) files and further imported into Cytoscape 3.8.0 for network topological analysis.

### 2.5. GO and KEGG Pathway Enrichment Analyses

The common targets were uploaded to the Database for Annotation, Visualization, and Integrated Discovery (DAVID) (https://davidbioinformatics.nih.gov/, version 6.8, accessed on 5 August 2025) for GO functional enrichment analysis to evaluate the targets in terms of biological process (BP), cellular component (CC), and molecular function (MF). In addition, KEGG pathway enrichment analysis was performed to investigate the potential signalling pathways involved in lactate action in MASLD and liver injury.

### 2.6. Molecular Docking

The 3D molecular structure of L-lactate (the biologically active enantiomer) was retrieved from the Traditional Chinese Medicine Systems Pharmacology Database and Analysis Platform (TCMSP)and exported in mol2 format. The structure was further optimized using Open Babel (v2.4.1) to correct bond orders, add missing hydrogen atoms, and generate the lowest-energy conformation. The SMILES string of L-lactate used in this study was C[C@H](O)C(=O)[O-].

High-resolution 3D crystal structures of the candidate target proteins were obtained from the RCSB Protein Data Bank (RCSB PDB; http://www.rcsb.org, accessed on 12 August 2025). The selection criteria for protein structures were as follows: resolution ≤ 2.5 Å; intact protein backbone and side chains; no missing residues in the ligand-binding domain; no non-specific co-crystallized ligands or additives.

Protein preprocessing was carried out using AutoDockTools 1.5.6: All water molecules and non-essential co-crystallized ligands were removed. Polar hydrogen atoms were added to the protein structure. Non-polar hydrogen atoms were merged. Gasteiger atomic charges were assigned to all atoms. The protein structure was saved in PDBQT format. Molecular docking simulations were performed using AutoDock Vina 1.1.2. Grid maps were generated using the AutoGrid module, with the grid box centered on the predicted ligand-binding pocket of each target protein. The exhaustiveness parameter was set to 16 to ensure comprehensive sampling of the binding space. All other parameters were kept at their default values. The optimal docking conformations and intermolecular interaction patterns were visualized using PyMOL 2.2.0.

### 2.7. MD Simulation

Molecular dynamics simulations were performed using GROMACS 2022, with the Amber99sb-ildn force field for target proteins and the General Amber Force Field (GAFF) for lactate. The TIP3P water model was used to solvate the lactate–protein complex system in a 10 × 10 × 10 nm cubic water box, with a 1.2 nm buffer between the protein and the box edges; the solvated system was neutralized by ion addition for automatic equilibrium. Long-range electrostatic interactions were calculated via the Particle-mesh Ewald (PME) method, and energy minimization was conducted using the steepest descent algorithm with 50,000 minimization steps. The cutoff distances for Coulombic and van der Waals interactions were both set to 1 nm. The system was subsequently equilibrated through two consecutive steps: the NVT (constant number, volume, temperature) ensemble and the NPT (constant number, pressure, temperature) ensemble. MD simulations were then carried out for 100 ns at a physiological temperature of 37 °C and atmospheric pressure (1 bar). The non-bonded interaction cutoff value was set to 10 Å. A Langevin thermostat was used to maintain the simulation temperature at 300 K, and the Berendsen barostat was applied for pressure control at 1 bar. After simulation, the root mean square deviation (RMSD), root mean square fluctuation (RMSF), and radius of gyration (Rg) of the complex were calculated to evaluate the stability of the lactate-protein binding.

### 2.8. Cell Culture, Differentiation and Treatment

AML12 mouse normal hepatocytes were purchased from Wuhan Punosai Biological Technology Co., Ltd. (Wuhan, China; Cat. No. CL-0602). Cells were authenticated by short tandem repeat (STR) profiling and confirmed to be free of mycoplasma contamination before use. Cells were maintained in DMEM/F12 supplemented with 10% (*v*/*v*) FBS, 0.5% (*v*/*v*) 100× ITS-G, 40 ng/mL dexamethasone and 1% (*v*/*v*) P/S. Cells were cultured at 37 °C in a humidified incubator containing 5% CO_2_.

Cells were randomly divided into the following six groups: Control group (C), cells cultured in complete DMEM/F12 medium only; Lactate control group (C + L), cells treated with 15 mM L-lactate; Steatotic AML12 cells model group (M), cells treated with 2.25 mM FFA mixture (1.5 mM OA + 0.75 mM PA); LDHA inhibitor group (M + Oxa), cells pre-treated with 25 μM sodium oxamate for 3 h, followed by treatment with a 1.5 mM mixture of free fatty acids and sodium oxalate; Lactate intervention group (M + L), cells treated with 2.25 mM free fatty acids (FFA) for 24 h to induce steatosis, followed by treatment with 15 mM L-lactate for another 24 h; PPARγ inhibitor group (M + L + GW9662), cells pre-treated with 3.3 nM GW9662 for 3 h, followed by treatment with other processing.

Sodium oxamate (HY-W013032A, MedChemExpress, Shanghai, China ) was dissolved in sterile H_2_O to prepare a 400 mM stock solution; GW9662 (MedChemExpress, HY-16578) was dissolved in DMSO to prepare a 10 mM stock solution; Lactate (MedChemExpress, HY-Y0479) was dissolved in sterile H_2_O to prepare a 400 mM stock solution. The concentrations of FFA, L-lactate, sodium oxamate, and GW9662 were determined based on preliminary dose–response experiments conducted in our laboratory and the published literature.

### 2.9. Ldha and CD36 siRNA Transfection and Knockdown Assay

Mouse CD36-specific siRNAs, Ldha-specific siRNA and scrambled negative control siRNA were synthesized by GenePharma (Shanghai, China). siCD36-1: 5′-GGAUGACAACUUCACAGUUTT-3′; siCD36-2: 5′-GGAUUGGAGUGGUGAUGUUTT-3′; siLdha-1: 5′-CCGAGUAAUUGGAAGUGGUTT-3′; siLdha-2: 5′-GCAAGAAGUGGAUGAAGAUTT-3′; negative control (siNC): 5′-UUCUCCGAACGUGUCACGUTT-3′. AML12 cells were transfected with siRNA using the siRNA-mate plus Transfection Kit (GenePharma Co., Ltd., Shanghai, China) when they reached 60% confluence, following the manufacturer’s standard protocols. Knockdown efficiency was verified by qRT-PCR 24 h after transfection.

### 2.10. Oil Red O Staining

Cells were cultured to approximately 70% confluence, fixed in 4% paraformaldehyde for 10 min, and washed twice with phosphate-buffered saline (PBS). Cells were then permeabilized in 60% isopropanol for 15 s, stained with Oil Red O working solution for 30 min, and quickly rinsed with 60% isopropanol for 15 s to remove excess dye. After three washes with distilled water, nuclei were counterstained with hematoxylin solution at room temperature for 1 min. Cells were rinsed twice more with distilled water, treated with bluing reagent for 5 s, washed again, and finally immersed in PBS for light microscopic visualization.

For semi-quantitative analysis, stained lipid droplets were extracted by incubating cells with 500 μL isopropanol at 37 °C for 10 min. A 100 μL aliquot was transferred to a 96-well plate, and absorbance was measured at 510 nm using a microplate spectrophotometer.

### 2.11. Animal Experiments

Male C57BL/6J mice (6-week-old, body weight 18–22 g) were obtained from Henan Skibbes Biotechnology Co., Ltd. (Zhengzhou, China). All animal protocols were reviewed and approved by the Animal Ethics Committee of Shenyang Agricultural University (approval No. SNLL25092902) with the approval date being 29 September 2025, and all procedures were performed in strict accordance with the National Institutes of Health Guide for the Care and Use of Laboratory Animals. Mice were maintained in a temperature-controlled (22 ± 2 °C), humidity-controlled (40–60%) SPF barrier facility under a strict 12 h light/12 h dark cycle (lights on at 7:00 AM), with free access to sterile food and water throughout the experiment. The experimental unit was the individual mouse. Mice were housed 4–5 per cage, and all experimental treatments were administered individually to each animal. Cage was included as a random effect in statistical models to control for potential cage effects. The reported *n* represents the number of individual mice per group.

After 1 week of acclimation, mice were randomized into two main groups: a normal diet control group (n = 39) and a high-fat diet (HFD) model group (n = 50). The control group was fed a low-fat diet (10% kcal from fat), while the HFD group received a 60% kcal high-fat diet (Research Diets, Cat. No. D12492), Table 1. Viral interventions were performed via tail vein injection at week 8. Mice received 4 × 10^11^ vector genomes (vg) of either AAV8-negative control virus (NC) or AAV8-LDHA knockdown virus (LDHA-KD). The all mice were further randomized into 8 experimental groups: C: Low-fat diet + saline injection (n = 13); C-NC: Low-fat diet + AAV8-NC injection (n = 13); C-LDHA: Low-fat + AAV8-shLDHA injection (n = 13), M: HFD + saline injection (n = 10); ML: HFD + saline injection (n = 10); M-NC: HFD + AAV8-NC injection (n = 10); M-LDHA: HFD + AAV8-shLDHA injection (n = 10); ML-LDHA: HFD + AAV8-shLDHA injection (n = 10). The randomisation sequence was generated using a computerised random number generator, and group assignment was performed by a research member who was not involved in the subsequent experimental interventions.

The knockdown efficiency was verified at week 4 (the 12th week of the experiment). From the C, C-NC, and C-LDHA groups, three animals were randomly selected from each group. Liver tissues were analyzed using EGFP fluorescence imaging and qPCR techniques to preliminarily validate the AAV8-mediated LDHA gene knockdown efficiency. Upon confirmation of successful knockdown, mice in the ML and ML-LDHA groups received 1.5 g/kg body weight/day of sodium L-lactate via oral gavage once daily between 9:00 AM and 10:00 AM for 4 weeks (from week 12 to week 16). Mice in all other groups received an equal volume of sterile saline as vehicle control.

Body weight was measured weekly throughout the experiment. At week 16, after an overnight fast (12 h), mice were anesthetized with 50 mg/kg pentobarbital sodium via intraperitoneal injection. Blood samples were collected from the orbital sinus for biochemical analysis. Liver tissues were harvested, weighed, and either fixed in 4% paraformaldehyde for histological analysis or snap-frozen in liquid nitrogen and stored at −80 °C. All inclusion and exclusion criteria for experimental animals and data points were established a priori prior to the start of the study. Inclusion criteria: Healthy 8-week-old male C57BL/6J mice with body weight ranging from 20 to 22 g. Exclusion criteria: Animals that died accidentally during the experiment, developed severe unrelated diseases, or failed to meet the model establishment criteria. Data points with obvious technical errors (e.g., sample contamination, instrument failure) were excluded from the final analysis. No animals died or were excluded during the entire experiment, resulting in a final sample size of n = 10 per group for all analyses.

### 2.12. AAV8 Vector Packaging and Titer Determination

#### 2.12.1. Plasmid Construction

The hepatocyte-specific AAV8-shRNA plasmid was constructed under the control of the thyroxine-binding globulin (TBG) promoter. The experimental plasmid (AAV8-TBG>Kozak-EGFP-mLdha[miR30-shRNA]-Poly(A)) encoded a miR30-based short hairpin RNA (shRNA) targeting mouse Ldha (NCBI Gene ID: 16828; Transcript ID: NM_010699.2). The control plasmid (AAV8-TBG>Kozak-EGFP-Poly(A)) carried only the enhanced green fluorescent protein (EGFP) reporter gene without any shRNA sequence.

#### 2.12.2. Cell Transfection

HEK293T cells were co-transfected with the target plasmid and two helper plasmids (pHelper and pRCVector) using Lipofectamine 3000 reagent (Thermo Fisher Scientific, Waltham, MA, USA)according to the manufacturer’s instructions.

#### 2.12.3. Virus Harvest

Culture supernatants and cell lysates were collected 48–72 h post-transfection, and pooled for subsequent viral purification.

#### 2.12.4. Virus Purification

Recombinant AAV8 vectors were purified via iodixanol density gradient ultracentrifugation, followed by buffer exchange and concentration using ultrafiltration centrifugal units.

#### 2.12.5. Viral Titer Assay

Viral titers were determined by quantitative PCR (qPCR) targeting the EGFP reporter gene. Titers were expressed as genome-containing viral particles per milliliter (vg/mL).

### 2.13. Real-Time Quantitative PCR (RT-qPCR)

Cells from each experimental group were collected, and total RNA was isolated using a commercial RNA extraction kit (Vazyme, Nanjing, China, No. RC112-01). After reverse transcription into cDNA, mRNA levels of target genes were quantified by RT-qPCR. Amplification was performed under the following conditions: initial denaturation at 95 °C for 1 min; 45 cycles of denaturation at 95 °C for 15 s and annealing/extension at 63 °C for 25 s, with fluorescence signals collected at each cycle. Each sample was analyzed in technical triplicate. Primer sequences are listed below (Table 2):

### 2.14. Western Blotting (WB)

Total protein was extracted using RIPA lysis buffer supplemented with 1% (*v*/*v*) PMSF and 1% (*v*/*v*) phosphatase inhibitor. The cells were scraped and lysed on ice for 30 min (with vortexing every 5 min); approximately 50 mg of liver tissue was homogenized on ice using a glass homogenizer and processed under the same conditions. All samples were centrifuged at 4 °C and 12,000× *g* for 20 min, and the supernatant was collected. Protein concentrations were determined using the BCA kit (Epizyme, Shangha, China, no ZJ101L) according to the instructions. All samples were diluted with RIPA buffer to achieve uniform concentration for consistent loading volumes. Add 5× SDS loading buffer (Shanghai Epizyme Biomedical Technology Co., Ltd., Shanghai, China, No LT101L) in a 1:4 volume ratio, mix thoroughly, and denature at 100 °C under dry bath for 10 min. After cooling, aliquot the solution and store at −80 °C, avoiding repeated freeze–thaw cycles. Protein samples were separated by 10% sodium dodecyl sulfate–polyacrylamide gel electrophoresis (SDS-PAGE) and transferred onto polyvinylidene difluoride (PVDF) membranes via cold wet transfer. Membranes were blocked with protein-free rapid blocking buffer at room temperature for 30 min and then incubated overnight at 4 °C with primary antibodies against the following targets: LDHA (dilution, 1:1000; cat. no. T55348S; Abmart, Shanghai, China), LDHB (dilution, 1:1000; cat. no. PS19376S; Abmart, Shanghai, China), CD36 (dilution, 1:800; cat. no. T55796S; Abmart, Shanghai, China), PPARγ (dilution, 1:800; cat. no. AF6284; Affinity, Changzhou, Jiangsu, China), β-actin (dilution, 1:1000; cat. no. ab822682; Abcam, Cambridge, UK). This was followed by incubation with the HRP*Goat Anti Rabbit IgG (dilution, 1:10,000; cat. no. RS0002; Immunoway, Suzhou, China) at room temperature for 1 h. Protein bands were visualised using chemiluminescent detection. The grayscale values were quantified using the ImageJ software (version 1.53t; National Institutes of Health, Bethesda, MD, USA).

### 2.15. Statistical Analysis

All statistical analyses were performed using IBM SPSS Statistics 25 and GraphPad Software, La Jolla, CA, USA). Normality of data distribution was assessed using the Shapiro–Wilk test. Normally distributed data are expressed as mean ± standard error of the mean (SEM) and compared using the unpaired two-tailed Student’s *t*-test. Non-normally distributed data, including all transcriptomic data from GEO datasets, are presented as box plots (median, IQR, and min/max values) and compared using the non-parametric Mann–Whitney U test. A *p*-value less than 0.05 was considered statistically significant. Statistical significance was denoted as * *p* < 0.05, ** *p* < 0.01, *** *p* < 0.001, and **** *p* < 0.0001.

## 3. Results

### 3.1. LDH Expression Is Upregulated in MASLD Patients and Mice

**Figure 1:**
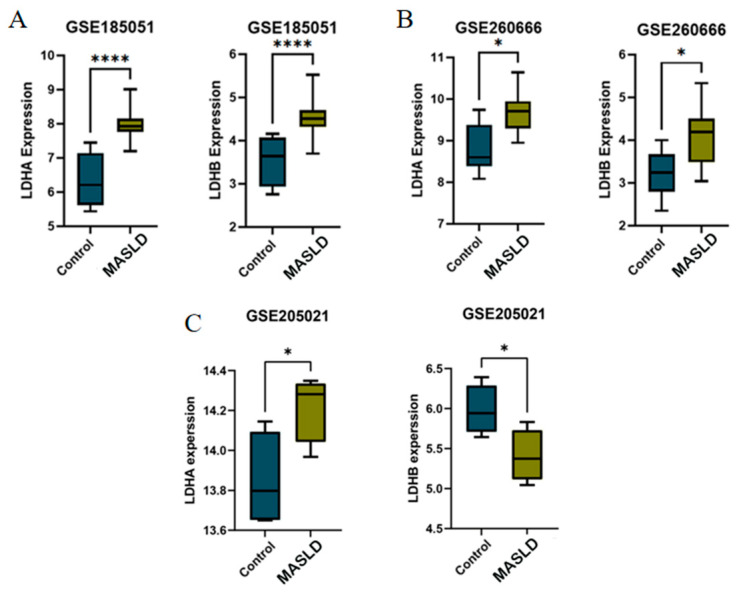
Upregulated hepatic LDHA and LDHB transcript expression in human and mouse MASLD. (**A**) Log_2_-transformed normalized read counts of LDHA and LDHB in liver tissues from the primary human validation cohort, consisting of 5 healthy controls and 52 biopsy-proven MASLD patients. (**B**) Log_2_-transformed normalized read counts of LDHA and LDHB in liver tissues from an independent adult Chinese cross-validation cohort, consisting of 6 healthy controls and 10 biopsy-proven MASLD patients. (**C**) Log_2_-transformed normalized read counts of LDHA and LDHB in liver tissues from a public mouse transcriptome dataset, consisting of 6 normal chow-fed control mice and 6 high-fat diet-induced MASLD model mice. Data are shown as box plots (median, interquartile range, and min/max values). Statistical significance was determined by Mann–Whitney U test. * *p* < 0.05, **** *p* < 0.0001 vs. control.

We first analyzed publicly available Gene Expression Omnibus (GEO) datasets to assess the mRNA expression profiles of lactate dehydrogenase isoforms LDHA and LDHB, the key enzymes regulating lactate homeostasis, in MASLD. Our analysis revealed that hepatic LDHA and LDHB mRNA levels were significantly elevated in MASLD patients compared with healthy control subjects (Figure 1A,B). Consistently, hepatic LDHA and LDHB expression were also significantly increased in MASLD mice relative to control mice (Figure 1C).

### 3.2. Revealing the Potential Targets of Lactate-MASLD by Using Network Pharmacology

**Figure 2:**
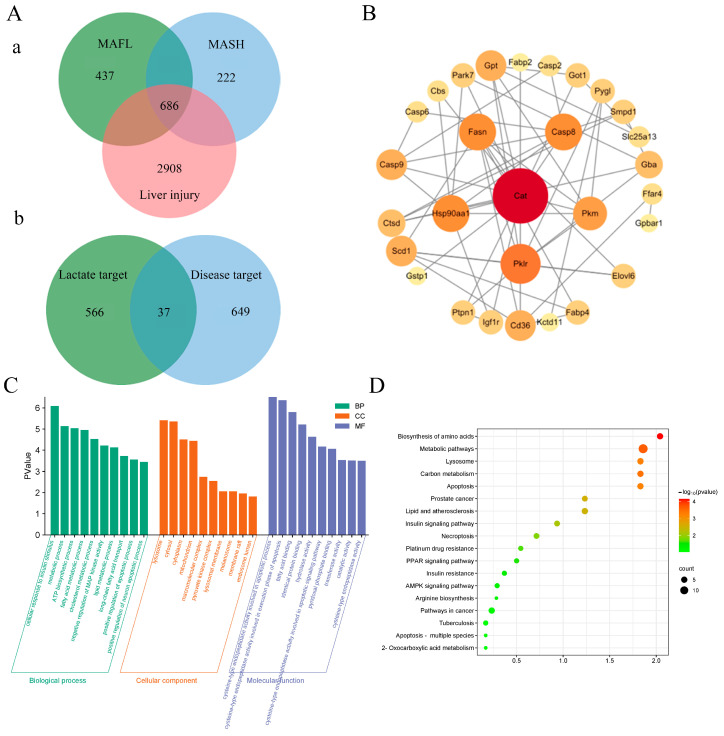
Identification of core therapeutic targets of lactate against MASLD spectrum disorders and functional enrichment analysis. (**A**) Venn diagram analysis. (a) Intersection of disease-related targets among MASLD, MASH, and liver injury. (b) Intersection of lactate-related targets and the above-identified common disease targets. (**B**) Protein–protein interaction (PPI) network of the 37 overlapping targets. Nodes represent target proteins, and edges represent experimentally validated interactions between proteins. The size and color depth of nodes are positively correlated with the degree value (larger and darker nodes indicate higher connectivity). Core hub genes were screened using the following criteria: betweenness centrality > 0.9, closeness centrality ≥ 0.27, and degree ≥ 7. (**C**) Gene Ontology (GO) enrichment analysis of the common targets, including biological process (BP, green), cellular component (CC, orange), and molecular function (MF, blue) categories. The bar length represents the −log_10_(*p*-value) of each enriched term. (**D**) Kyoto Encyclopedia of Genes and Genomes (KEGG) pathway enrichment analysis of the common targets. The color of the bubbles represents the −log_10_(*p*-value) (red indicates higher statistical significance), and the size of the bubbles represents the number of enriched genes in the corresponding pathway. All bioinformatics analyses were performed using R software (version 4.3.1) and the STRING database (version 12.0).

A total of 602 non-redundant potential targets of lactate action were identified after this rigorous filtering process using the following databases: SwissTargetPrediction, TCMSP, PharmMapper Service, and ChEMBL. The search results for the keywords “non-alcoholic fatty liver disease,” “simple fatty liver disease,” “non-alcoholic steatohepatitis,” and “liver injury” were analyzed in the GeneCards, OMIM, Disgenet, PHARMGKB, DrugBank, and NCBI databases. Duplicate entries were removed, resulting in a total of 686 non-redundant MASLD and liver injury-related targets (Figure 2A, a). The collected targets were uploaded to the online platform of Microbiology (http://www.bioinformatics.com.cn/), and a Venn diagram with 37 intersected targets was generated (Figure 2A, b). We then constructed a network graph using Cytoscape 3.8.0 to illustrate the relationship between lactate and MASLD. Furthermore, protein–protein interaction (PPI) analysis based on three biological parameters (betweenness interaction score > 0.9, closeness centrality ≥ 0.27, and degree ≥ 7) allowed us to select the top 10 predicted target genes with the highest association values (Table 3).

**Figure 3:**
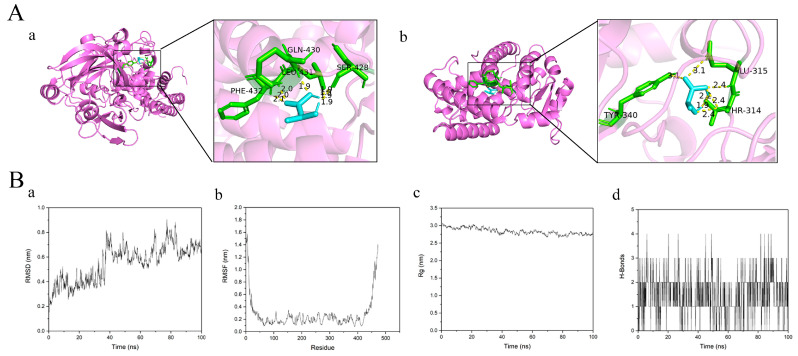
Molecular docking analysis and molecular dynamics (MD) simulation validation of the interaction between lactate and core therapeutic targets. (**A**) Three-dimensional (3D) molecular docking models. (a) Binding mode of lactate with CD36. (b) Binding mode of lactate with PPARγ. Green sticks represent the key amino acid residues lining the binding pocket, cyan sticks represent the ligand lactate, and yellow dashed lines represent hydrogen bonds with the labeled bond lengths in angstroms (Å). (**B**) MD simulation analysis of the conformational stability of the lactate–CD36 complex over a 100 ns production run. (a) Root mean square deviation (RMSD) of the CD36 protein backbone as a function of simulation time, reflecting the overall structural stability of the complex. (b) Root mean square fluctuation (RMSF) of each residue in the CD36 protein backbone, indicating the local flexibility of different protein regions. (c) Time evolution of the radius of gyration (Rg) of the CD36 protein, evaluating the overall compactness of the protein structure. (d) Temporal distribution of the number of hydrogen bonds formed between lactate and CD36 during the entire simulation process.

The overlapping targets were imported into the Database for Annotation, Visualization and Integrated Discovery (DAVID) for Gene Ontology (GO) functional enrichment analysis (Figure 3A). Biological Process (BP) enrichment results were primarily associated with metabolic processes, ATP biosynthesis, fatty acid metabolism, cholesterol metabolism, lipid metabolism, and long-chain fatty acid transport. Cellular Component (CC) enrichment results were mainly enriched in the plasma membrane, cytoplasm, lipid droplet, and endoplasmic reticulum, which are key subcellular structures involved in lipid metabolism. Molecular Function (MF) enrichment results were predominantly related to fatty acid binding, catalytic activity, and cysteine-type endopeptidase activity involved in apoptosis.

Kyoto Encyclopedia of Genes and Genomes (KEGG) pathway enrichment analysis (Figure 3B) revealed that the overlapping targets were significantly enriched in metabolic pathways, lipid and atherosclerosis, PPAR signaling pathway, and insulin resistance. These computational findings suggest that lactate may participate in MASLD progression by regulating lipid metabolism-related signaling pathways. Given that the lactate receptor GPR81 has been previously reported to modulate PPARγ expression, we selected PPARγ and CD36 as the key candidate molecules for subsequent functional validation based on the overlapping target gene functions and KEGG enrichment results.

### 3.3. Molecular Docking and MD Simulations

We performed molecular docking simulations using AutoDock Vina to investigate potential interactions between lactate and the candidate proteins PPARγ and CD36. In general, a binding affinity of ≤−4.25 kcal/mol is considered a threshold for potential binding interactions, ≤−5.0 kcal/mol indicates favorable predicted binding, and ≤−7.0 kcal/mol suggests high predicted binding affinity [14]. Our docking results showed that lactate exhibited predicted binding affinities of −4.5 kcal/mol for CD36 (PDB ID: 5LGD) and −4.2 kcal/mol for PPARγ (PDB ID: 1WMO), respectively, suggesting possible weak interactions between lactate and both proteins (Figure 3A). Given that CD36 was prioritized as a key candidate target in our network pharmacology analysis, we further conducted molecular dynamics (MD) simulations to evaluate the dynamic stability of the lactate–CD36 complex. To evaluate the binding stability and conformational dynamics of the lactate–CD36 complex, 100 ns all-atom molecular dynamics simulations were performed, and root-mean-square deviation (RMSD), radius of gyration (Rg), root-mean-square fluctuation (RMSF), and intermolecular hydrogen bonds were systematically analyzed (Figure 3B). MD simulation results showed that the root-mean-square deviation (RMSD) of the CD36 main chain increased rapidly during the initial simulation phase and reached dynamic equilibrium after 20 ns, with stable fluctuations ranging from 0.2 to 0.9 nm and no significant drift. These findings indicate favorable kinetic convergence and conformational stability of the lactate–CD36 complex. The radius of gyration (Rg) values remained stable between 2.7 and 3.0 nm with a mild downward trend throughout the simulation trajectory, suggesting that the overall folding and spatial structure of CD36 remained compact and stable without unfolding or conformational loosening. Root-mean-square fluctuation (RMSF) analysis revealed high flexibility in the N- and C-terminal regions of CD36, whereas residues within the core functional domain and ligand-binding pocket exhibited low RMSF values and high conformational rigidity, which further supports the predicted stable binding of lactate to CD36. Furthermore, sustained intermolecular hydrogen bonds were predicted between lactate and CD36 throughout the 100 ns MD simulation trajectory. The number of hydrogen bonds fluctuated steadily with no prolonged interruptions, and these interactions likely contribute to anchoring lactate within the CD36 ligand-binding pocket and may help maintain the thermodynamic and kinetic stability of the complex. Collectively, these computational data support the possibility of a direct interaction between lactate and CD36, as evidenced by the favorable conformational convergence, compact overall structure, and persistent intermolecular interactions observed in the simulations.

### 3.4. Elevated Lactate Levels in Steatotic AML12 Cells and Culture Supernatants

**Figure 4:**
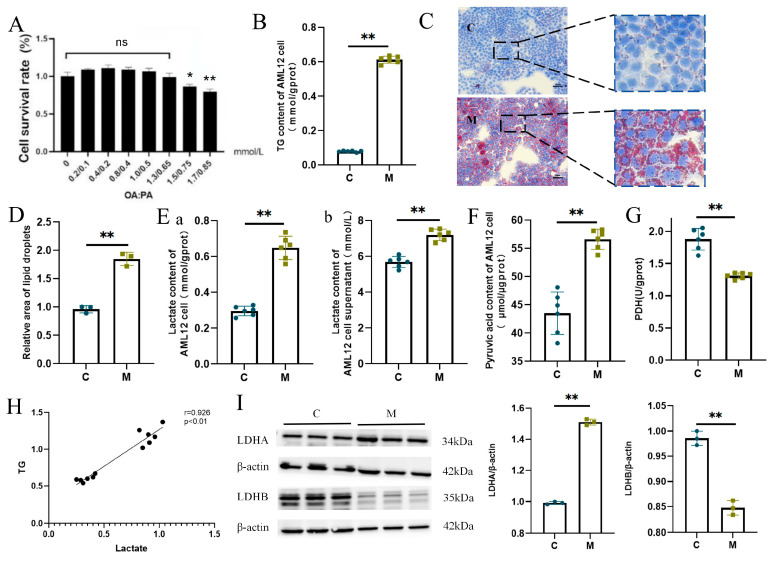
AML12 steatosis model establishment and lactate metabolism characterization. (**A**) CCK-8 assay of AML12 cell viability after 24 h treatment with gradient concentrations of OA/PA (2:1, *v*/*v*) (n = 6). (**B**) Intracellular triglyceride (TG) content in control (C) and model (M) groups (n = 6). (**C**) Oil Red O staining of AML12 cells (200×, n = 3). (**D**) Semi-quantification of lipid droplet area (n = 3). (**E**) Intracellular (a) and supernatant (b) lactate levels (n = 6). (**F**) Intracellular pyruvate content (n = 6). (**G**) Pyruvate dehydrogenase (PDH) activity (n = 6). (**H**) Pearson correlation between intracellular TG and lactate levels (n = 8, r = 0.926, *p* < 0.01). (**I**) Western blot analysis and quantification of LDHA and LDHB protein expression (β-actin as loading control, n = 3 independent experiments). Data are presented as mean ± SEM. Statistical comparisons were performed using unpaired two-tailed Student’s *t*-test. NS, not significant; * *p* < 0.05; ** *p* < 0.01 vs. control.

To validate our in vivo findings of dysregulated lactate metabolism in MASLD, we established an in vitro hepatocellular steatosis model using AML12 mouse hepatocytes treated with an oleic acid/palmitic acid (OA:PA = 2:1, *v*/*v*) mixture. The CCK-8 assay identified 2.25 mM total fatty acid (1.5 mM OA + 0.75 mM PA) as the maximum non-cytotoxic concentration for subsequent experiments (Figure 4A). Oil Red O staining confirmed successful steatosis induction: control cells showed minimal lipid accumulation, whereas steatotic cells exhibited extensive cytoplasmic lipid droplet deposition (Figure 4C). Semi-quantitative analysis revealed a significant increase in lipid droplet area in model cells (Figure 4D), accompanied by a significant elevation in intracellular triglyceride (TG) levels (Figure 4B). Steatotic AML12 cells exhibited markedly higher intracellular and supernatant lactate levels than control cells (Figure 4E, a and b). Meanwhile, intracellular pyruvate levels (Figure 4F) and pyruvate dehydrogenase (PDH) activity (Figure 4G) were significantly increased, indicating enhanced glycolytic flux under steatotic conditions. Pearson correlation analysis revealed a robust positive correlation between intracellular TG and lactate levels (r = 0.826, *p* < 0.01, Figure 4H). Western blot analysis identified altered expression of core lactate-metabolizing enzymes: LDHA expression was markedly increased, whereas LDHB expression was suppressed in steatotic AML12 cells (Figure 4I).

### 3.5. Lactate Exacerbates Steatosis in AML12 Cells

**Figure 5:**
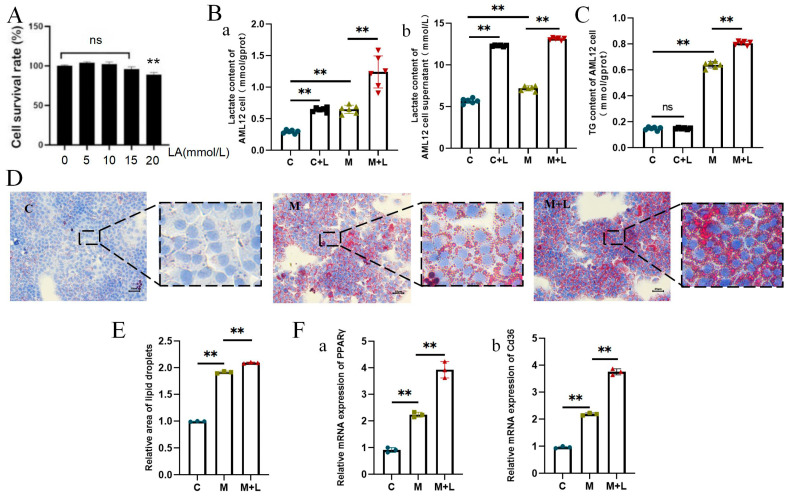
Exogenous lactate exacerbates steatosis in AML12 hepatocytes. (**A**) CCK-8 assay of AML12 cell viability after 24 h treatment with gradient concentrations of lactate (n = 6). (**B**) Intracellular (a) and supernatant (b) lactate levels in the four groups (n = 6). (**C**) Intracellular triglyceride (TG) content (n = 6). (**D**) Oil Red O staining of AML12 cells (200×, n = 3). The Oil Red O staining result of the C + L group is provided in Supplementary Figure S1. (**E**) Semi-quantification of relative lipid droplet area (n = 3). (**F**) RT-qPCR analysis of PPARγ (a) and CD36 (b) mRNA expression (n = 3). Data are presented as mean ± SEM. Statistical comparisons were performed using one-way ANOVA followed by Tukey’s post hoc test. ns, not significant; ** *p* < 0.01.

To determine the non-cytotoxic concentration of exogenous lactate, we performed a Cell Counting Kit-8 (CCK-8) assay. Treatment with 15 mM lactate had no significant effect on AML12 cell viability, whereas 20 mM lactate significantly reduced cell viability (Figure 5A). Accordingly, 15 mM lactate was selected for all subsequent experiments. Cells were divided into four experimental groups: control (C), control treated with 15 mM lactate (C + L), oleic acid/palmitic acid (OA/PA)-induced steatosis model (M), and steatosis model treated with 15 mM lactate (M + L). Exogenous lactate treatment significantly increased lactate levels in both intracellular and supernatant fractions of control and steatotic cells (Figure 5B). Notably, intracellular triglyceride (TG) levels were comparable between the C and C + L groups but were significantly elevated in the M group and further increased in the M + L group (Figure 5C), indicating that exogenous lactate treatment was associated with enhanced lipid accumulation specifically in steatotic hepatocytes.

Consistently, Oil Red O staining revealed minimal lipid droplets in control cells, whereas extensive cytoplasmic lipid deposition was observed in M cells, and this deposition was further augmented by lactate treatment (Figure 5D). The representative Oil Red O staining image of the C + L group is provided in Supplementary Figure S1, which confirmed that lactate treatment alone did not induce obvious lipid droplet formation in normal AML12 cells, consistent with the intracellular TG results. Since the core focus of this experiment was to verify the exacerbation effect of lactate on established hepatocyte steatosis, semi-quantitative analysis of lipid droplet area and detection of steatosis-related gene expression were performed focusing on the steatotic cell model (Figure 5E,F). RT-qPCR analysis further showed that the mRNA expression levels of *Pparg* and *Cd36* were significantly upregulated in the M group, and these upregulations were further enhanced by exogenous lactate treatment (Figure 5F).

### 3.6. Lactate Exacerbates Lipid Deposition in AML12 Cells via the PPARγ/CD36 Signaling Axis

**Figure 6:**
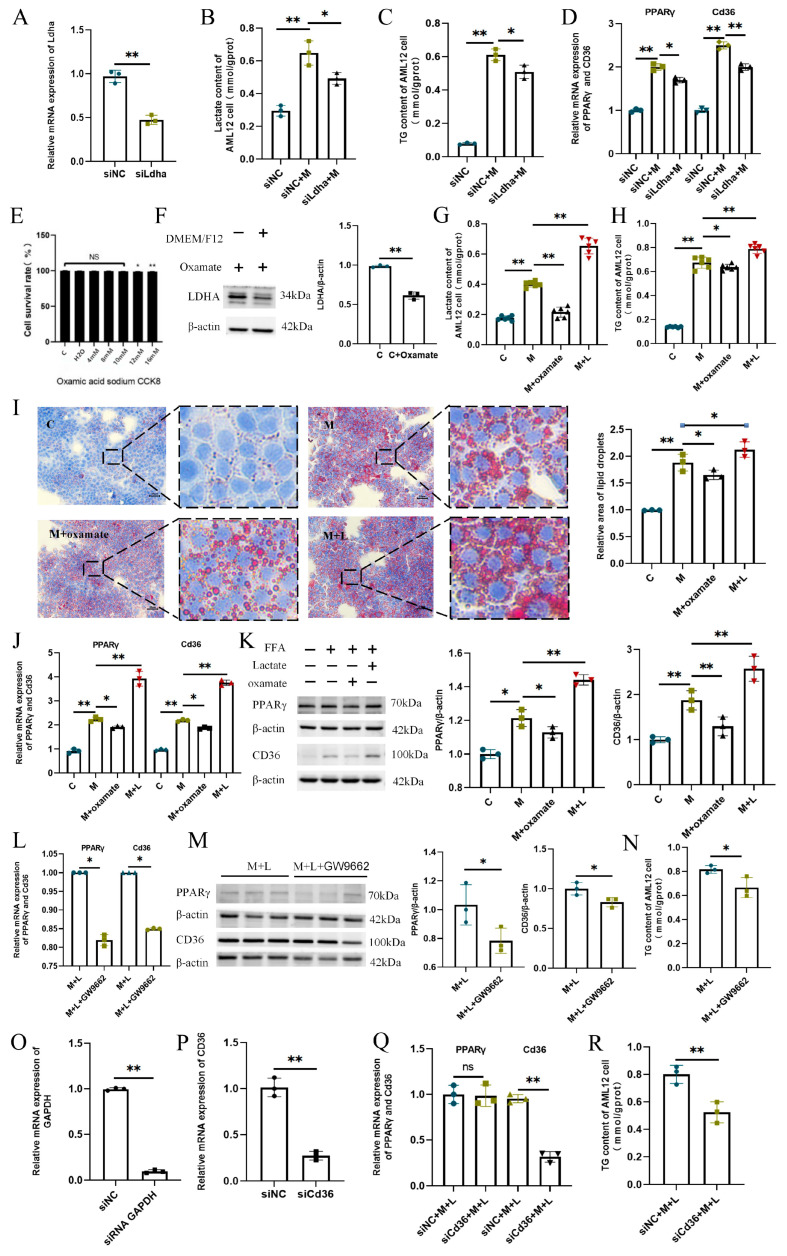
Lactate promotes hepatocyte steatosis via the PPARγ/CD36 signaling axis. (**A**) RT-qPCR analysis of Ldha mRNA expression after transfection with siNC and siLdha (n = 3). (**B**) Intracellular lactate levels in the groups (n = 3). (**C**) Intracellular triglyceride (TG) content (n = 3). (**D**) RT-qPCR analysis of PPARγ and CD36 mRNA expression (n = 3). (**E**) CCK-8 assay of AML12 cell viability after 24 h treatment with gradient concentrations of sodium oxamate (n = 6). (**F**) Western blot analysis and quantification of LDHA protein expression (β-actin as loading control, n = 3). (**G**) Intracellular lactate levels in the four groups (n = 6). (**H**) Intracellular triglyceride (TG) content (n = 6). (**I**) Oil Red O staining of AML12 cells and Semi-quantification of relative lipid droplet area (n = 3). (**J**) RT-qPCR analysis of PPARγ and CD36 mRNA expression (n = 3). (**K**) Western blot analysis and quantification of PPARγ and CD36 protein expression (β-actin as loading control, n = 3). (**L**) RT-qPCR analysis of PPARγ and CD36 mRNA expression (n = 3). (**M**) Western blot analysis and quantification of PPARγ and CD36 protein expression in M + L and M + L + GW9662 groups (β-actin as loading control, n = 3). (**N**) Intracellular TG content in M + L and M + L + GW9662 groups (n = 3). (**O**) The GAPDH mRNA levels were analyzed using RT-qPCR to verify the efficiency of the transfection reagent (n = 3). (**P**) RT-qPCR analysis of CD36 mRNA expression after transfection with siNC and siCD36 (n = 3). (**Q**) RT-qPCR analysis of PPARγ and CD36 mRNA expression (n = 3). (**R**) Intracellular triglyceride (TG) content (n = 3). Data are presented as mean ± SEM. Statistical comparisons were performed using one-way ANOVA followed by Tukey’s post hoc test. ns, not significant; * *p* < 0.05; ** *p* < 0.01.

qPCR results showed that transfection with Ldha-specific siRNA (siLdha) significantly reduced Ldha mRNA levels by approximately 50% (Figure 6A). The knockdown efficiency of LDHA was verified at both the mRNA and protein levels. Transfection with siLDHA resulted in approximately 50% reduction in both LDHA transcript and protein expression compared with the negative control (Figure 6A, Supplementary Figure S2), which was used for subsequent functional experiments. Based on these findings, we used an FFA mixture (OA:PA = 2:1) to induce steatosis in AML12 cells. Our results showed that lactate levels in the siNC + M group were significantly higher than those in the siNC group, while Ldha knockdown significantly reversed the abnormal lactate accumulation in steatotic cells (Figure 6B). In contrast, intracellular triglyceride (TG) levels were significantly elevated in steatotic cells, whereas Ldha knockdown significantly reduced TG levels in steatotic cells (Figure 6C). qPCR results showed that *Pparg* and *Cd36* mRNA expression levels were significantly elevated in steatotic AML12 cells, whereas Ldha knockdown significantly suppressed the abnormal upregulation of these two genes (Figure 6D). The CCK-8 assay identified 10 mM oxamate as the maximum non-cytotoxic concentration (Figure 6E). Oxamate treatment significantly reduced LDHA protein expression (Figure 6F) and intracellular lactate levels in steatotic AML12 cells (Figure 6G). Importantly, inhibition of lactate production by oxamate significantly decreased intracellular triglyceride (TG) levels (Figure 6H) and lipid droplet accumulation (Figure 6I) in steatotic cells. Conversely, exogenous lactate treatment further exacerbated these lipid accumulation effects. RT-qPCR and Western blot analysis showed that oxamate treatment significantly reduced the mRNA and protein expression of *PPARγ* and *CD36*, which were elevated in steatotic cells and further increased by exogenous lactate (Figure 6J,K). Matched NaCl controls with pH adjustment showed no significant effect on lipid accumulation, confirming that the observed effects were due to lactate and oxamate rather than changes in sodium concentration or pH (Supplementary Figure S3).

We employed GW9662, a specific PPARγ antagonist, to confirm the requirement of PPARγ for lactate-induced steatosis. As a direct downstream target gene of PPARγ, CD36 is widely used to reflect the transcriptional activity of PPARγ pathway in hepatic lipid metabolism studies. In this study, the elevation of PPARγ and its downstream target CD36 induced by exogenous lactate was significantly reduced by 3.3 nM GW9662 treatment at both mRNA and protein levels (Figure 6L,M). The increase in intracellular TG levels induced by lactate treatment was consistently reversed by GW9662 treatment (Figure 6N). To further confirm the role of CD36 as a downstream effector in this pathway, we knocked down Cd36 expression in AML12 cells using siRNA. qPCR results showed that transfection with Cd36-specific siRNA (siCd36) significantly reduced Cd36 mRNA levels by approximately 70% (Figure 6O,P). Knockdown of cellular Cd36 expression followed by stimulation with FFA and lactate did not affect Pparg expression. However, it reversed the lactate-induced increase in intracellular TG levels (Figure 6R).

### 3.7. AAV8-TBG-shLdha Reduces Hepatic Ldha Expression In Vivo

**Figure 7:**
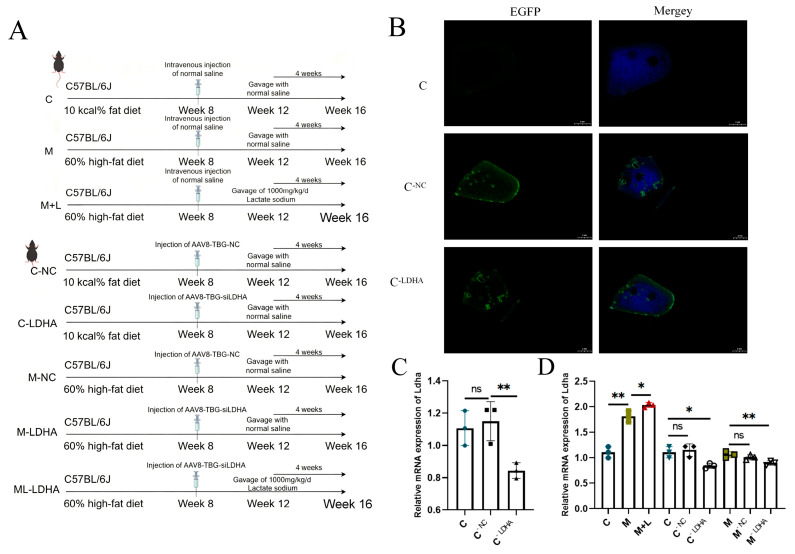
Validation of AAV8-mediated hepatic Ldha knockdown in mice. (**A**) Schematic diagram of the experimental design and treatment timeline for all animal groups. (**B**) EGFP fluorescence imaging of liver tissues at week 12. Scale bar = 2 mm (representative images from 3 independent mice per group). Green: EGFP; blue: DAPI nuclear staining. (**C**) Relative Ldha mRNA expression in liver tissues at week 12 (n = 3). (**D**) Relative Ldha mRNA expression in liver tissues at week 16 (n = 3). Data are presented as mean ± SEM. Statistical comparisons were performed using one-way ANOVA followed by Tukey’s post hoc test. ns, not significant; * *p* < 0.05; ** *p* < 0.01.

The experimental design and treatment timeline for all in vivo experiments are illustrated in Figure 7A. To evaluate the hepatic transduction efficacy and long-term stability of the recombinant adeno-associated viruses, mice were intravenously injected with AAV8-TBG-NC or AAV8-TBG-shLdha via the tail vein at week 8. EGFP fluorescence imaging of liver tissues harvested at week 12 demonstrated broad, liver-specific transduction in all AAV-injected groups, whereas untreated control mice exhibited no detectable fluorescence (Figure 7B). At week 12, quantitative reverse transcription-polymerase chain reaction (qRT-PCR) analysis revealed that hepatic Ldha mRNA expression was significantly reduced in both the C-LDHA and M-LDHA groups compared with their respective negative control (NC) groups (Figure 7C). This reduction persisted until the end of the experiment at week 16 (Figure 7D).

### 3.8. Lactate Supplementation Is Associated with Aggravated Hepatic Steatosis and Increased PPARγ/CD36 Expression in Mice

**Figure 8:**
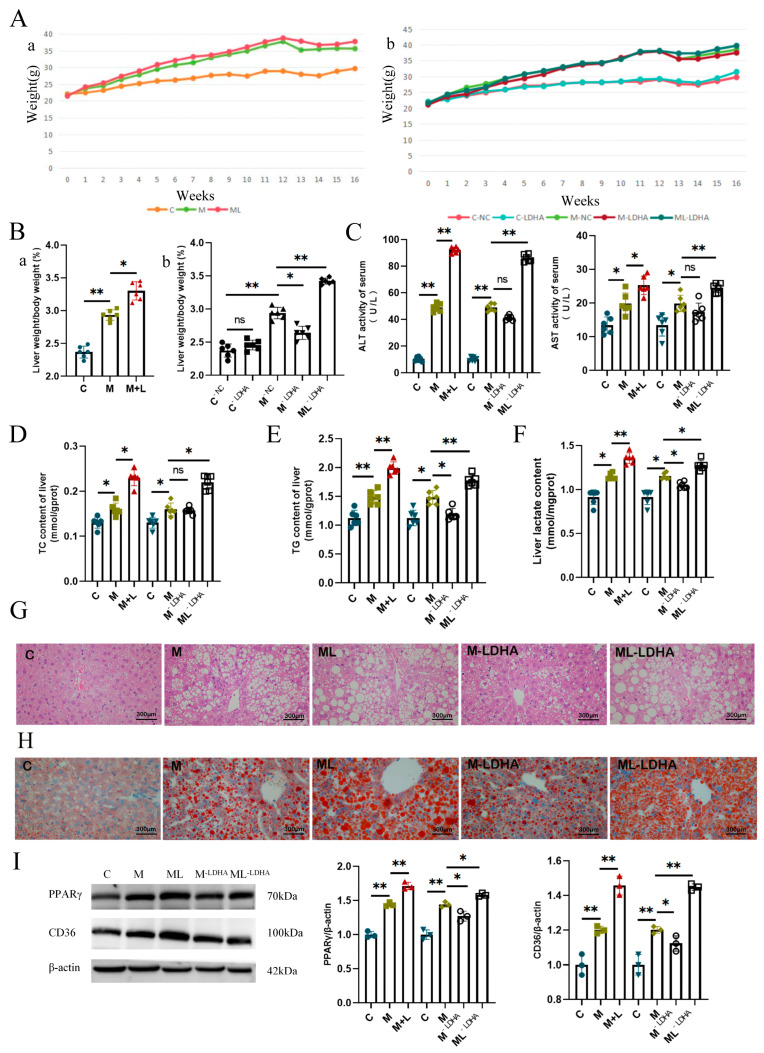
Lactate promotes MASLD progression via PPARγ/CD36 axis in vivo. (**A**) Body weight changes of mice in different groups over 16 weeks (n = 6). (a) Body weight trajectory of normal control mice; (b) Body weight trajectory of adenovirus-injected mice. (**B**) Liver-to-body weight ratio at week 16 (n = 6). (**C**) Serum ALT and AST activities at week 16 (n = 6). (**D**) Hepatic total cholesterol (TC) content (n = 6). (**E**) Hepatic triglyceride (TG) content (n = 6). (**F**) Hepatic lactate content (n = 6). (**G**) Hematoxylin and eosin (H&E) staining of liver sections (200×, n = 6). (**H**) Oil Red O staining of liver frozen sections (200×, n = 6). (**I**) Western blot analysis and quantification of hepatic PPARγ and CD36 protein expression (β-actin as loading control, n = 3). Data are presented as mean ± SEM. Statistical comparisons were performed using one-way ANOVA followed by Tukey’s post hoc test. ns, not significant; * *p* < 0.05; ** *p* < 0.01.

We next investigated whether our in vitro findings could be recapitulated in a high-fat diet (HFD)-induced MASLD mouse model, using a combination of exogenous lactate supplementation and liver-specific Ldha knockdown. HFD feeding resulted in a progressive and significant increase in body weight over the 16-week experimental period (Figure 8A). Consistently, the liver-to-body weight ratio (Figure 8B) and serum activities of alanine aminotransferase (ALT) and aspartate aminotransferase (AST) (Figure 8C) were markedly elevated in HFD-fed mice, accompanied by significant increases in hepatic total cholesterol (TC), triglyceride (TG), and lactate concentrations (Figure 8D–F). Mice that received exogenous lactate supplementation exhibited further significant elevations in all these metabolic and liver injury parameters. In contrast, liver-specific Ldha knockdown significantly attenuated HFD-induced liver injury and lipid accumulation. Histological analysis using hematoxylin and eosin (H&E) and Oil Red O staining demonstrated that lactate-supplemented mice displayed more severe hepatic steatosis, inflammatory cell infiltration, and lipid droplet deposition, whereas Ldha knockdown ameliorated these histological abnormalities (Figure 8G,H). Western blot analysis revealed that hepatic PPARγ and CD36 protein expression levels were increased in HFD-fed mice, further upregulated in the lactate-supplemented group, and downregulated in the Ldha-knockdown group (Figure 8I).

## 4. Discussion

Metabolic dysfunction-associated steatotic liver disease (MASLD) is a prevalent chronic liver disorder affecting approximately 150 million individuals globally and accounting for nearly 2 million deaths annually. Abnormal hepatic lipid deposition is the primary initiating factor that triggers and drives disease progression. Under pathological stress, hepatocyte metabolism shifts from oxidative phosphorylation to aerobic glycolysis, accompanied by elevated lactate dehydrogenase (LDH) activity and progressive hepatic lactate accumulation [15,16,17,18,19,20]. Clinical evidence has linked serum lactate levels and LDH activity to the prognosis of liver failure and hepatocellular carcinoma (HCC) [21], supporting a critical role of lactate in the pathogenesis of chronic liver diseases. This study aimed to elucidate the regulatory effect and molecular mechanism of lactate on hepatic steatosis, to provide new mechanistic insights and potential intervention targets for MASLD.

LDH is a tetrameric enzyme composed of LDHA and LDHB subunits, with LDHA preferentially catalyzing pyruvate-to-lactate conversion and LDHB favoring the reverse reaction. By analyzing three Gene Expression Omnibus (GEO) datasets (GSE260666, GSE185051, and GSE205021), we identified consistent upregulation of the hepatic LDHA/LDHB ratio in both MASLD patients and mouse models, despite divergent absolute LDHB expression across cohorts. This isoenzyme shift determines the overall catalytic direction of LDH toward lactate production, which was further validated at the protein level in oleic acid/palmitic acid (OA/PA)-induced steatotic AML12 cells. A transcriptomic meta-analysis by Sandeep et al. [22] (datasets GSE83452 and GSE61260) also demonstrated enhanced hepatic LDHA enzyme activity in metabolic dysfunction-associated steatohepatitis (MASH) and high-fat diet (HFD)-induced mouse steatosis, which is highly concordant with our findings.

We further revealed the metabolic basis underlying this catalytic shift: steatotic hepatocytes exhibited elevated pyruvate levels and impaired pyruvate dehydrogenase (PDH) activity. As the rate-limiting enzyme linking glycolysis to the tricarboxylic acid (TCA) cycle, reduced PDH activity blocks pyruvate entry into mitochondrial oxidative phosphorylation, leading to intracellular pyruvate accumulation. In the context of enhanced glycolytic flux, accumulated pyruvate is preferentially converted to lactate by LDHA [23]. This process is further reinforced by the HIF-1α/PDK1 positive feedback loop widely reported in MASLD: activated HIF-1α upregulates both LDHA and PDK1 transcription, and PDK1 inactivates PDH via phosphorylation, thereby amplifying glycolytic reprogramming and lactate production [24]. Together, these findings delineate a complete metabolic cascade from glycolysis activation to excessive lactate generation in MASLD.

To explore the specific mechanism by which lactate modulates MASLD progression, we employed network pharmacology approaches combined with molecular docking and molecular dynamics simulations to prioritize candidate targets and generate mechanistic hypotheses. By analyzing the overlapping targets associated with lactate and MASLD, we identified a panel of putative core hub targets exhibiting high connectivity. These core targets are implicated in critical biological processes such as oxidative stress responses, lipid metabolism regulation, and metabolic reprogramming. PKLR and PKM1/PKM2 are critical enzymes in the glycolytic pathway, whereas ELOVL6, FASN, IGF1R, FABP2, PYGL, AOX1, and CD36 serve as key regulators of lipid metabolism. Among these, CD36 is a member of the class B scavenger receptor family and serves as a critical fatty acid transporter; it mediates increased uptake of free fatty acids by hepatocytes, promotes excessive hepatic lipid accumulation, and transduces pro-inflammatory signals in the liver. CD36 is widely recognized as a critical regulator of MASLD progression [25]. Multiple clinical studies have shown a positive correlation between hepatic CD36 levels and the severity of lipid deposition across the MASLD spectrum [26,27,28,29,30].

Gene Ontology (GO) enrichment analysis revealed that lactate-regulated targets were predominantly enriched in three main domains: biological processes (lipid metabolism, fatty acid metabolism, and long-chain fatty acid transport), cellular components (cytoplasm, mitochondria, macromolecular complexes, and lipid rafts), and molecular functions (fatty acid binding and catalytic activity). Kyoto Encyclopedia of Genes and Genomes (KEGG) pathway analysis further indicated that these targets were significantly enriched in lipid metabolism-related pathways, such as lipid metabolism, lipid and atherosclerosis, PPAR signaling pathway, and insulin resistance. The PPARγ/CD36 signaling axis is a canonical pathway regulating hepatic lipid metabolism [31]. PPARγ is highly expressed in metabolic organs and serves as a critical regulator of systemic lipid metabolism, lipogenesis, and insulin sensitivity [32,33,34,35,36]. CD36 (also known as fatty acid translocase) is a transmembrane glycoprotein that primarily mediates the cellular uptake and intracellular trafficking of long-chain fatty acids. As a critical regulator of free fatty acid metabolism, CD36 is critically involved in hepatic lipid homeostasis, inflammatory cascades, and liver fibrosis progression [37,38,39].

Accordingly, we performed molecular docking to explore potential interactions between lactate and PPARγ/CD36, and the results suggested favorable binding poses between lactate and these candidate receptors. Subsequent molecular dynamics simulations of the lactate–CD36 complex exhibiting the highest docking score further supported the conformational stability of this predicted complex under simulated physiological conditions. We further compared the binding region of lactate with the canonical orthosteric ligand-binding pocket (LBP) of PPARγ to evaluate potential competitive interaction with natural ligands. Canonical PPARγ agonists, including endogenous polyunsaturated fatty acids and synthetic thiazolidinediones, bind deep within the Y-shaped hydrophobic LBP and form a conserved hydrogen bond network with residues Ser289, His323, His449 and Tyr473, which is critical for stabilizing the active conformation of helix 12 (AF-2) and recruiting coactivators [40]. In our molecular docking model, lactate mainly binds to the entrance region of the PPARγ ligand-binding domain, with no spatial overlap with the deep orthosteric ligand-binding site. This result indicates that there is no direct competitive binding between lactate and canonical PPARγ ligands. Instead, lactate may fine-tune the conformational dynamics and transcriptional activity of PPARγ through an allosteric effect at the pocket entrance, which is consistent with the property of lactate as an endogenous metabolic mediator exerting mild regulatory effects. Collectively, these in silico findings suggest that lactate may regulate hepatic lipid metabolism, at least in part, through the PPARγ/CD36 signaling axis and contribute to MASLD progression. Notably, as lactate is a small endogenous metabolite, target prediction databases may generate non-specific or low-confidence associations.

A series of in vitro and in vivo functional assays were performed to verify this hypothesis. In FFA-induced steatotic AML12 cells, intracellular lactate levels were positively correlated with triglyceride (TG) accumulation. Exogenous lactate supplementation exacerbated lipid deposition and upregulated PPARγ/CD36 expression, whereas both pharmacological LDHA inhibition by sodium oxamate and genetic LDHA knockdown (siLDHA) [41,42] ameliorated steatosis and reversed the activation of the PPARγ/CD36 axis. Furthermore, blockade of PPARγ activity by its specific antagonist GW9662 significantly attenuated lactate-induced lipid accumulation, confirming that PPARγ activity is indispensable for the lipogenic effect of lactate.

We further performed CD36-specific loss-of-function experiments. We subjected AML12 cells with CD36 gene knockdown (siCD36) to combined FFA and lactate treatment, and the results showed that CD36 knockdown reversed the lactate-induced exacerbation of steatosis in AML12 cells. Consistent with our findings, a previous study by Tang et al. [43] showed that arsenic exposure in mice reduced hepatic PDHA1 activity, leading to increased conversion of pyruvate to lactate. Accumulated intracellular lactate in hepatocytes induced site-specific lactylation of histone H3 at lysine 18 (H3K18la), which was significantly enriched at the CD36 promoter region. This epigenetic modification directly enhanced CD36 transcriptional activity, promoted fatty acid uptake, and activated NLRP3 inflammasomes. LDHA knockdown was shown to inhibit arsenic-induced CD36 upregulation. These findings suggest that histone lactylation may represent a potential epigenetic mechanism underlying lactate-mediated CD36 regulation. However, this mechanism was not explored in the present study, and further validation is needed to determine whether it contributes to lactate-induced hepatic steatosis in the context of MASLD.

In vivo, we established a MASLD mouse model using a widely used high-fat diet feeding protocol; this model recapitulates key pathological features and disease progression of human MASLD. Concurrently, we developed a liver-specific LDHA knockdown mouse model via tail vein injection of adeno-associated virus serotype 8 (AAV8) expressing TBG promoter-driven shLDHA (AAV8-TBG-shLDHA). This experimental design allowed us to investigate the role of lactate in MASLD pathogenesis in vivo using two complementary approaches: exogenous lactate supplementation and liver-specific genetic knockdown of LDHA. Systemic LDHA inhibition is associated with significant cardiac, muscular, and cerebral toxicity, whereas liver-specific delivery systems (including AAV8 gene therapy, GalNAc-conjugated siRNAs, and OATP-targeted small molecules) significantly improve the therapeutic safety profile. Martinez-Turrillas et al. [44] employed an AAV8 vector to deliver the CRISPR-Cas9 system and achieved liver-specific knockout of the LDHA gene. Following a single intravenous administration, hepatic LDHA activity was reduced by more than 90%. Whole-genome sequencing revealed no detectable off-target effects, and no significant hepatotoxicity or systemic toxicity was observed in the treated mice. Exogenous lactate administration aggravated hepatocellular steatosis, elevated hepatic TC and TG levels, and increased serum ALT/AST activities in MASLD mice, accompanied by upregulation of the PPARγ/CD36 axis; conversely, liver-specific LDHA knockdown via AAV8-TBG-shLDHA exerted the opposite protective effect. Collectively, complementary pharmacological and genetic evidence strongly supports that LDHA-dependent lactate production promotes hepatic steatosis via the PPARγ/CD36 signaling axis. It should be noted that our study does not confirm a direct physical interaction between lactate and PPARγ, nor does it exclude the involvement of intermediate signaling molecules. Luciferase reporter assays, chromatin immunoprecipitation (ChIP) and in vivo rescue experiments are required to elucidate the precise molecular mechanism.

In addition to the direct PPARγ/CD36 pathway and epigenetic regulation mentioned above, hepatic circadian clock regulation may represent another important indirect regulatory mechanism underlying lactate-exacerbated hepatic steatosis [45,46,47]. It is well established that the circadian clock acts as a master upstream regulator of hepatic lipid homeostasis, and it directly controls the transcription of PPAR family members as well as their downstream target genes including Cd36 at the rhythmic level. Perturbation of circadian clock function has been widely demonstrated to disrupt lipid metabolic homeostasis and drive the development and progression of chronic liver diseases including MASLD and liver fibrosis, through synergistically dysregulating metabolic, inflammatory and TGF-β signaling pathways. Notably, a recent study confirmed that hepatic TGF-β signaling is under direct circadian clock control, and pharmacological targeting of the circadian clock effectively prevents liver fibrosis by restoring the rhythmic homeostasis of TGF-β signaling [48].

As a core glycolytic metabolite with intrinsic diurnal fluctuation, lactate has been reported to interfere with the normal oscillation of core clock genes in hepatocytes by modulating cellular redox state and NAD+-dependent deacetylase activity [49,50,51,52]. Therefore, we speculate that accumulated lactate in the steatotic liver may not only directly activate the PPARγ/CD36 axis but also dysregulate hepatic circadian clock function, which in turn amplifies the aberrant expression of PPARγ and CD36 and synergistically promotes hepatic lipid deposition. This potential circadian regulatory layer provides a broader mechanistic perspective for our findings, and the interplay between lactate metabolism and hepatic circadian clock in MASLD deserves further in-depth investigation in future studies.

### 4.1. Clinical Implications

Our findings carry important translational relevance for MASLD management. For risk stratification, hepatic and circulating lactate levels may serve as a candidate metabolic biomarker to identify high-risk individuals with progressive MASLD. For disease prevention, interventions targeting glycolytic flux to reduce hepatic lactate accumulation, such as optimized dietary patterns or regular aerobic exercise, may represent a feasible strategy to delay MASLD onset in susceptible populations. For therapeutic development, the LDHA–lactate–PPARγ/CD36 axis provides a set of novel intervention targets. Liver-specific LDHA inhibition or selective modulation of the PPARγ/CD36 axis may alleviate excessive hepatic lipid deposition, offering alternative therapeutic options for MASLD patients with limited response to current treatments.

Several limitations of this study should be acknowledged. First, all mechanistic conclusions are drawn from in vitro cell models and in vivo mouse experiments; independent clinical cohort validation is still needed to confirm the correlation between lactate levels and MASLD severity in humans. Second, this study focuses on the PPARγ/CD36-dependent pathway, and the contributions of parallel mechanisms such as histone lactylation and circadian regulation require further experimental verification. Third, Metabolic dysfunction-associated steatotic liver disease (MASLD) is a highly complex, multifactorial disorder with a pathogenesis involving multiple interconnected pathophysiological pathways. Pathways independent of lactate also contribute to hepatic lipid accumulation, and no single factor can fully explain the complex pathogenic mechanisms of MASLD. Building on our prior network pharmacology analyses, this study highlights the lactate–PPARγ–CD36 axis as a candidate mediator implicated in the pathogenesis and progression of MASLD. Notably, this axis represents only one potentially important pathway among the numerous mechanisms underlying MASLD and does not explain the full spectrum of molecular networks involved in hepatic lipid metabolism regulation.

### 4.2. Limitations

Several limitations of this study should be acknowledged. First, as a mechanistic investigation, our findings are derived primarily from cellular and murine models, and validation in independent human patient cohorts with diverse ethnic backgrounds is limited to public GEO datasets. Larger prospective clinical cohorts are needed to confirm the prognostic value of the LDHA/LDHB ratio in human MASLD. Second, while we demonstrate the functional necessity of the PPARγ/CD36 axis, the precise molecular mechanism by which lactate modulates this pathway—including direct receptor binding, epigenetic regulation via histone lactylation, or intermediate signaling mediators—requires further definitive investigation. Third, the circadian clock hypothesis proposed herein remains speculative and has not been experimentally validated in our MASLD model systems. Finally, MASLD is a multifactorial disorder driven by multiple interconnected pathophysiological pathways; the lactate–PPARγ–CD36 axis represents only one contributing mechanism and does not fully account for the full complexity of hepatic lipid dysregulation in vivo.

Building on these findings, our ongoing work includes integrated transcriptomic and metabolomic analyses of steatotic hepatocytes to map the full molecular network regulated by lactate. Future studies will formally test the circadian clock hypothesis and explore epigenetic mechanisms such as histone lactylation to delineate the complete signaling cascade linking lactate to CD36 upregulation. In vivo rescue experiments and clinical cohort validation will further establish the translational potential of targeting the lactate–PPARγ–CD36 axis for MASLD management.

## 5. Conclusions

This study integrates network pharmacology analysis with in vitro and in vivo experimental models and employs intervention strategies including LDHA inhibition and targeted genetic knockdown to investigate the pathogenic role and characterize the putative molecular mechanisms of lactate in metabolic dysfunction-associated steatotic liver disease (MASLD). Our results indicate that elevated lactate levels are positively correlated with MASLD progression and the upregulation of the PPARγ/CD36 signaling axis and suggest that lactate exacerbates hepatic lipid metabolism disorders at least in part through this axis. These findings highlight the LDHA–lactate–PPARγ/CD36 axis as a potential therapeutic target for MASLD and suggest that targeting this axis represents a promising direction for future mechanistic and translational research.

## Figures and Tables

**Table 1:** Nutritional composition table of feed ration.

Product	D12450J (10 kcal% Fat)		D12492 (60 kcal% Fat)	
	gm%	kca/%	gm%	kca/%
Protein	16.9	20.0	26.2	20.0
Carbohydrate	67.3	70.0	26.3	20.1
Fat	4.3	10.0	34.9	59.9
Total		100.0		100.0

**Table 2:** Fluorescence quantitative PCR primer sequence.

Gene	Primer Sequences	GenBank Accession Number
*Pparg*	F-CACTCGCATTCCTTTGAC R-TCGCACTTTGGTATTCTT	NM_001127330.3
*Cd36*	F-GCAGGTCTATCTACGCTGTGTTCG R-TGTCTGGATTCTGGAGGGGTGATG	NM_001159555.2
*Ldha*	F-CTGTGGCAGACTTGGCTGAGAG R-GGATACATGGGACACTGAGGAAGAC	NM_001136069.2
*Ldhb*	F-TGTGGTGGTGACGGCAGGAG R-AGGGCTGTACTTGACGATCTGAGG	NM_001302765.1
*Fasn*	F-TGCCCGAGTCAGAGAACCTACAG R-TCCATAGAGCCCAGCCTTCCATC	NM_007988.3
*Pklr*	F-TGTGGTGGCAGTCCGAGATG R-ACTTCTTCACGCCTTCATGGTT	NM_001099779.1
*Casp8*	F-GCATCCTGACTGGCGTGAAC R-AGGTGGGCTGTGGCATCTG	NM_001080126.2
*Pkm*	F-ACCATCAAGAATGTCCGTGAAGC R-TCCAGAGCCACCGCAACAG	NM_001253883.2
*β-Actin*	F-ACTGCCGCATCCTCTTCCTC R-AACCGCTCGTTGCCAATAGTG	NM_007393.5

**Table 3:** The top 10 target targets ranked by degree value.

Targets	PDB ID	Method	Resolution (Å)	R-Value Free	R-Value Work	R-Value Observed
CAT	3OVX	X-RAY DIFFRACTION	1.49	0.226	0.200	0.202
PKLR	4OG8	X-RAY DIFFRACTION	1.53	0.176	0.151	0.152
HSP90AA1	5J80	X-RAY DIFFRACTION	1.17	0.191	0.179	0.180
FASN	3TJM	X-RAY DIFFRACTION	1.48	0.224	0.190	0.192
CASP8	2VWR	X-RAY DIFFRACTION	1.30	0.177	0.134	0.136
PKM	7THI	X-RAY DIFFRACTION	1.33	0.160	0.134	0.136
CD36	5LGD	X-RAY DIFFRACTION	2.07	0.253	0.212	0.214
Casp9	3D9T	X-RAY DIFFRACTION	1.50	0.205	0.183	0.186
SCD1	4ZYO	X-RAY DIFFRACTION	3.25	0.264	0.241	0.242
Gpt	4TRB	X-RAY DIFFRACTION	2.4	0.286	0.228	0.231
PPARG	1WM0	X-RAY DIFFRACTION	2.90	0.295	0.191	0.195

## Data Availability

The raw data supporting the conclusions of this article will be made available by the authors on request.

## Supplementary Materials

The following supporting information can be downloaded at: https://www.mdpi.com/article/10.3390/cells15141240/s1, Figure S1: Oil Red O staining of normal AML12 cells with or without lactate treatment; Figure S2: Western blot analysis and quantification of LDHA protein expression; Figure S3: Matched sodium/osmolarity and pH controls confirm the specific effects of sodium oxamate and sodium lactate on intracellular triglyceride accumulation in steatotic AML12 cells.

## Abbreviations

AST	Aspartate aminotransferase
ALT	Alanine aminotransferase
CD36	Cluster of differentiation 36
HFD	High-fat diet
LDH	Lactate dehydrogenase
MD	Molecular dynamic
OA	Oleic acid
PA	Palmitic acid
PPARγ	Peroxisome proliferator-activated receptor gamma
TG	Triglyceride
