# Supplementary material for: Lactate Aggravates MASLD via PPARγ/CD36-Mediated Hepatocellular Fatty Acid Uptake
Source: Cells. 2026 Jul 9;15(14):1240. doi: 10.3390/cells15141240 (PMC13406211; doi:10.3390/cells15141240)
Supplement: Supplementary file 1 [file cells-15-01240-s001.zip › Supplementary Figures.pdf]

Supplementary Figures:

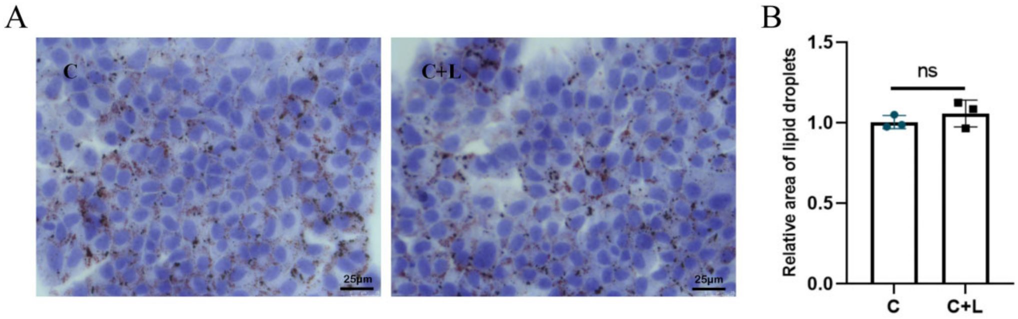

Supplementary Figure S1. Oil Red O staining of normal AML12 cells with or without lactate treatment. Representative images show no obvious lipid droplet accumulation in both control (C) and 15 mM lactate-treated (C+L) AML12 cells (×200, *n* = 3). Data are presented as mean ± SEM. Statistical comparisons were performed using Student's t-test. ns, not significant.

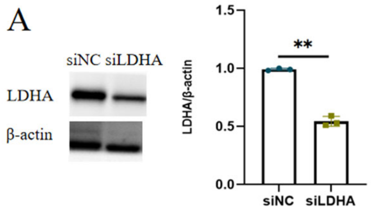

Supplementary Figure S2. Western blot analysis and quantification of LDHA protein expression (β-actin as loading control, *n*=3). Data are presented as mean± SEM. Statistical comparisons were performed using one-way ANOVA followed by Tukey's post-hoc test. **p < 0.01.

Reagents

Sodium oxamate: MedChemExpress, Cat# HY-W013032A

Sodium L-lactate: MedChemExpress, Cat# HY-Y0479

Sodium chloride (NaCl): Sigma-Aldrich, S7653

Sterile 1 M HCl and 1 M NaOH solutions for pH adjustment

Medium Preparation and pH Matching and Osmolality Calibration

AML12 complete medium was prepared and divided into five equal aliquots .

The following additions were made to each 11.25 mL base medium aliquot:

(1) Control group: 0.75 mL sterile ultrapure water

(2) Oxamate group: 0.3 mL 400 mM sodium oxamate + 0.45 mL sterile ultrapure water (final concentration: 10 mM)

(3) Oxamate-NaCl control group: 0.3 mL 400 mM NaCl + 0.45 mL sterile ultrapure water (final concentration: 10 mM)

(4) Lactate group: 0.45 mL 400 mM sodium L-lactate + 0.3 mL sterile ultrapure water (final concentration: 15 mM)

(5) Lactate-NaCl control group: 0.45 mL 400 mM NaCl + 0.3 mL sterile ultrapure water (final concentration: 15 mM)

All media were pre-warmed to 37°C in a water bath before pH measurement.

(1) The pH of each medium was measured using a calibrated precision ` pH meter (accuracy ±0.01) at 37°C.

(2) The pH of the Oxamate-NaCl control medium was adjusted to exactly match that of the Oxamate group using sterile 1 M HCl or 1 M NaOH.
(3) The pH of the Lactate-NaCl control medium was adjusted to exactly match that of the Lactate group using the same method.
(4) All media were measured for osmolality using a freezing-point osmometer (Osmomat 3000, Gonotec, Germany) at 25°C. The osmolality of all groups was adjusted to within ±5 mOsm/L of each other by adding small volumes of sterile 5 M NaCl or ultrapure water.
(5) All media were filter-sterilized through 0.22 μm filters before use.

Cell Treatment and Phenotypic Analysis

(1) AML12 cells were seeded in 6-well plates at a density of 2×10^5 cells per well and cultured for 24 hours until reaching 70% confluence.
(2) Hepatocellular steatosis was induced by treatment with OA/PA for 24 hours, as described in the main Materials and Methods section.
(3) The induction medium was completely removed, and cells were treated with 2 mL per well of the five different media prepared above.
(4) Intracellular lipid accumulation was assessed by quantitative triglyceride (TG) content measurement, following the protocols detailed in the main Materials and Methods section.

Result

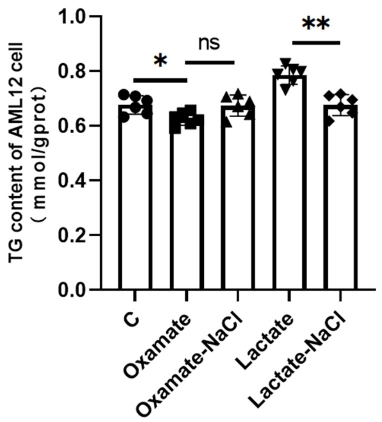

Supplementary Figure S3. Matched sodium/osmolarity and pH controls confirm the specific effects of sodium oxamate and sodium lactate on intracellular triglyceride accumulation in steatotic AML12 cells. Data are presented as mean ± SD (n = 6). Statistical analysis was performed using unpaired Student's t-test for pre-planned comparisons between specific groups, *$p < 0.05$, **$p < 0.01$, ns, not significant.
